# The Effects of Riboflavin/Ultraviolet: A Corneal Cross-Linking on the Signs and Symptoms of Bullous Keratopathy

**DOI:** 10.4103/0974-9233.75889

**Published:** 2011

**Authors:** Hamid Gharaee, Mohammad-Reza Ansari-Astaneh, Fatemeh Armanfar

**Affiliations:** Khatam-al-Anbia Eye Research Center, Mashhad University of Medical Science, Islamic Republic of Iran

**Keywords:** Bullous Keratopathy, Corneal Cross-Linking, Ultraviolet-A

## Abstract

**Purpose:**

To evaluate the effectiveness of corneal cross-linking in improving the signs and symptoms of bullous keratopathy.

**Materials and Methods:**

This prospective non-randomized case series evaluated 20 eyes with bullous ketratopathy that underwent corneal cross-linking (C3R) with riboflavin and ultraviolet-A (UVA, 370 nm, 3 mW/cm^2^). C3R was performed for 30 min in a routine procedure after removal of epithelium. Central corneal thickness (CCT), corneal haze, visual acuity (VA), and the presence of irritating symptoms were recorded before the procedure, and at 1 week, 1 months, 3 months, and 6 months after the procedure.

**Results:**

The mean CCT was 872 ± 162 μm (range: 665-1180 μm) before the procedure. Following the procedure, CCT was 855 ± 175 μm after 1 week, 839 ± 210 μm after 1 month, 866 ± 185 μm after 3 months, and 863 ± 177 μm after 6 months (*P*>0.05, all visits). There was no significant improvement in VA or corneal clarity after 6 months. Improvement of the following symptoms: burning, pain, and foreign body sensation were reported after 6 months by 83.3%, 75.0%, and 66.7% of patients, respectively. Persistent epithelial defect occurred in five patients (25%) resolved with frequent lubrication and bandage contact lenses.

**Conclusion:**

The outcomes of this study indicate corneal cross-linking is not an effective treatment for bullous keratopathy with respect to VA and CCT, although it can improve irritation and discomfort.

## INTRODUCTION

Bullous keratopathy is a visually-disabling corneal disorder caused by endothelial cell dysfunction. Without sufficient endothelial function, fluid accumulates in the extracellular spaces between collagen fibers and lamellae causing corneal swelling. Although treatment with a topical hypertonic solution temporarily improves corneal clarity, keratoplasty is the only treatment that will significantly improve visual acuity (VA). Corneal transplantation is not a good option in the case of poor visual potential. Therefore, other palliative modalities were introduced such as stromal puncture and bandage contact lenses in symptomatic cases.^1^,^2^

Collagen cross linking (C3R) is a photochemical treatment that has been introduced over the past 5 years to stabilize progressive keratoconus^3^ and postoperative laser *in situ* keratomileusis (LASIK) ectasia.^4^,^5^ Recently, riboflavin (0.1%) and ultraviolet-A (UVA) collagen cross-linking (C3R) has shown potential to improve the signs and symptoms of bullous keratopathy.^6^,^7^ Riboflavin and UV irradiance can strengthen corneal tissue by increasing collagen covalent bonds, similar to what happens in polymers. For bullous keratopathy, this may act to prevent water infiltration, reducing epithelial edema in the decompensated cornea. The objective of this study is to demonstrate the effects of C3R in improving the sign and symptoms of bullous keratopathy.

## MATERIALS AND METHODS

This prospective clinical trial was performed between February 2008 and April 2009 on 20 eyes of 20 subjects with bullous keratopathy. Follow up was performed at the Khatam-al-Anbia eye hospital, Masshad, Iran. Visual impairment of affected eyes in all subjects was related to corneal decompensation. Subjects with corneal scarring or other eye disease affecting VA were excluded. We evaluated the best corrected visual acuity (BCVA), central corneal thickness (CCT), corneal clarity index, and symptoms including pain, burning and foreign-body sensation before C3R, and again at 1 week, 1 month, 3 months, and 6 months after C3R. Riboflavin (0.1%) eye drops were applied every 3 min for 30 min after corneal epithelial removal (9 mm) and corneal cross-linking with riboflavin and UVA (370 nm, 3 mW/cm 2). Riboflavin drops were also applied every 5 min during C3R. Bandage contact lenses were removed after 1 week or complete epithelial healing. Chloramphenicol drops were prescribed every 6 h for 1 week. Central corneal thickness was measured with ultrasound pachymetry during each follow-up visit. Corneal clarity was graded by visual slit-lamp inspection according to a previously published 7corneal clarity score with the following ratings: 1 – very opaque cornea, difficult to visualize underlying structures; 2 – relatively opaque cornea with some visualization of underlying structures; 3 – diffuse corneal edema with relative clarity to visualize underlying structures; and 4 – clear cornea. Grading corneal clarity and symptoms was performed by one ophthalmologist who was blinded to the procedure or which follow-up visit was being assessed. All data were recorded in preformated data collection forms and the results were evaluated in two categories: signs (quantitative) and symptoms (qualitative). The Wilcoxon signed-rank tests evaluated the differences before and after the procedure. Written consent was obtained from all subjects and they were informed about treatment options and possible risks. This clinical trial was approved by the Review Board/Ethics Committee of the Mashhad University of Medical Sciences (MUMS) Eye Research Center.

## RESULTS

**Figure 1:**
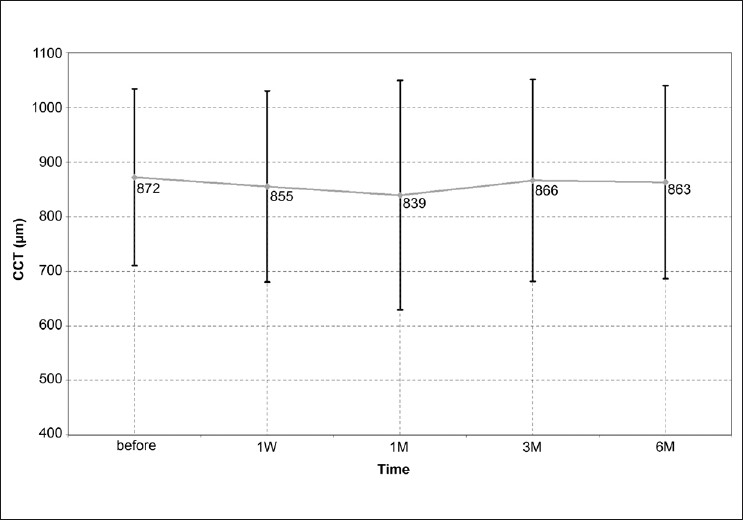
Central corneal thickness measured by ultrasound pachymetry before and after corneal cross-linking

**Figure 2:**
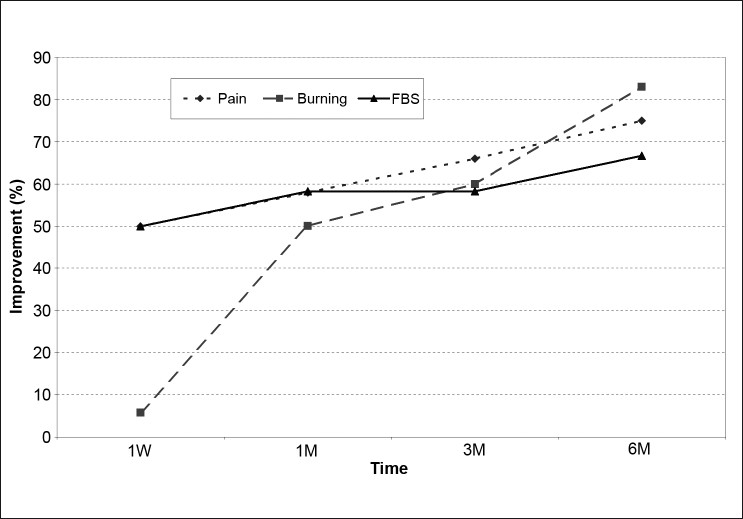
Improvement in the bullous keratopathy symptoms after corneal cross linking. Symptoms evaluated were burning, pain, and foreign body sensation at each follow up

**Figure 3:**
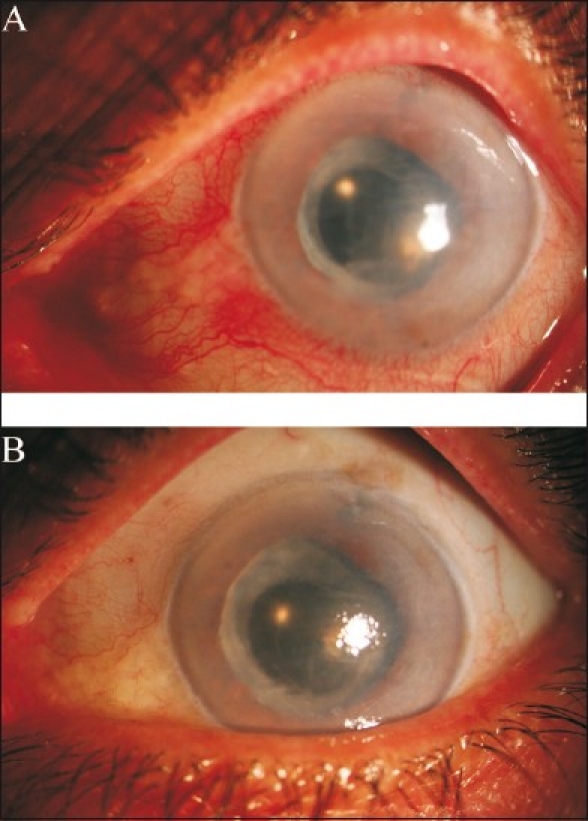
Slit photograph of a subject with bullous keratopathy. A: before and B: 6 months after corneal cross linking. There were less injection and the patient was symptom free 6 months after the procedure

The mean age of the cohort was 66.4 ± 8.2 years. The etiology of bullous keratopathy was pseudophakia in 11 eyes, aphakia in 4 eyes and failed graft in 5 eyes. The mean central corneal thickness was 872 ± 162μm before C3R and 855 ± 175, 839 ± 210, 866 ± 185 and 863 ± 177 μm at 1 week, 1 month, 3 months, and 6 months after C3R, respectively. There was no significant change in central corneal thickness at each follow up (*P*>0.05). The mean central corneal thickness was unchanged after 6 months [Figure 1]. VA was count fingers in all subjects preoperatively, and there was no improvement in VA (*P*>0.05). The corneal clarity remained statistically similar before and after C3R (*P*>0.05). Six months after the procedure there was an improvement in symptoms such as burning in 83% of the subject, pain in 75% of the subjects and foreign body sensation in 67% of the subjects [Figure 2]. Persistent epithelial defect occurred in five subjects (25%), and was resolved with frequent lubrication and bandage contact lens. No other significant complications were observed in the cohort [Figure 3].

## DISCUSSION

Patients with bullous keratopathy often present with symptoms that affect quality of life. Relieving symptoms for these patients is of the utmost importance, especially for patients that are ineligible for corneal grafts. Several therapies exist to treat bullous keratopathy and include bandage contact lenses, conjunctival flap, electro- cauterization, topical therapy with 5% saline and corneal graft.^1^,^2^ Riboflavin (0.1%) and UVA collagen cross-linking is a chemically reactive process that has been used in progressive keratoconus and post-LASIK ectasia. Stromal absorption of riboflavin is best achieved with repeated topical application after epithelial cell removal.

Krueger *et al*,^7^ reported the first case of C3R in bullous keratopathy prior to corneal transplantation with successful results. The corneal edema present in bullous keratopathy may limit penetration of riboflavin to the deep layers of cornea. Therefore, Krueger *et al*,^7^ used two consecutive corneal pockets (350 and 150 μm deep) that were created using a femtosecond laser and sequential intrastromal injections of 0.1% riboflavin (0.2 ml) were performed before UVA irradiation.^7^ Krueger *et al*,^7^ reported improvement of VA and decreased corneal thickness which differs from the results of our study. This difference may be due to differing riboflavin application techniques between studies. As femtosecond lasers are not widely available, we could not perform Krueger *et al*,^7^ procedure. Additionally the potential exists for additional complication by creating two pockets in the corneal stroma.

Wollensak *et al*,^7^ showed that C3R may be useful in treating bullous keratopathy, reporting improvements in VA and symptoms after C3R. Wollensak *et al*, used a procedure similar to ours, but their sample size (3 patients) was not large enough to evaluate the effect of C3R on parameters such as visual acuity or CCT.

A previous study^8^ evaluated the therapeutic effect of C3R on the symptoms of bullous keratopathy and indicated that there are likely benefits to using C3R to treat the symptoms of bullous keratopathy. However, the follow-up period in the Gadelha *et al*,^8^ study was short (2 months) and similar to our study, they also found no significant improvement in VA or a decrease in CCT after 2 months.

Studies reporting the use of C3R to treat bullous keratopathy are rare.^6^–^8^ To date, our study is the largest case study and has the longest follow-up time in English peer-reviewed literature. We evaluated 20 patients, which allowed us to perform relatively robust statistical analyses to evaluate the effects of C3R on bullous keratopathy. In our study, the subjects were followed out to 6 month which appears to be sufficient to confirm the effectiveness of the procedure.

This difference between our results and other smaller case studies may be due to different criteria of patient selection or differential usage of riboflavin. Patients in the early stages of the disease, without any scarring or large bulla, may respond better to this type of therapy,^9^ but this has yet to be demonstrated and should be the subject of future studies. Anterior stromal puncture is a simple and low-cost option that requires only a slit-lamp and needle. Comparatively, C3R is not a cost-effective method to relieve the irritating symptoms of bullous keratopathy. The success rate of anterior stromal puncture for relieving pain is 65%, which is similar to the C3R procedure.^1^ Accordingly, we would not recommend C3R in symptomatic patients with bullous keratopathy as anterior stromal puncture may be a better option in these patients. Due to the high rate of recurrent erosions as an important complication and no improvement in VA, we do not recommend C3R for treating bullous keratopathy.

Presentation at a meeting: 19^th^ Annual Congress of the Iranian Society of Ophthalmology
